# Real-world treatment patterns, discontinuation and clinical outcomes in patients with B-cell lymphoproliferative diseases treated with BTK inhibitors in China

**DOI:** 10.3389/fimmu.2023.1184395

**Published:** 2023-07-07

**Authors:** Yuting Yan, Rui Lv, Tingyu Wang, Ying Yu, Yanshan Huang, Wenjie Xiong, Yuxi Li, Weiwei Sui, Qi Wang, Wenyang Huang, Gang An, Dehui Zou, Jianxiang Wang, Lugui Qiu, Shuhua Yi

**Affiliations:** ^1^State Key Laboratory of Experimental Hematology, National Clinical Research Center for Blood Diseases, Haihe Laboratory of Cell Ecosystem, Institute of Hematology & Blood Diseases Hospital, Chinese Academy of Medical Sciences & Peking Union Medical College, Tianjin, China; ^2^Tianjin Institutes of Health Science, Tianjin, China

**Keywords:** treatment patterns, adverse events, BTK inhibitor, discontinuation, outcome

## Abstract

**Introduction:**

Bruton tyrosine kinase inhibitor (BTKi) has demonstrated substantial efficacy in treating B-cell lymphoproliferative diseases (BLPD). Nonetheless, the significant discontinuation rates due to toxicity or financial reasons cannot be overlooked. In China, empirical evidence on the usage of BTKi remains scarce.

**Methods:**

To address this, a retrospective cohort study was conducted focused on 673 Chinese patients with BLPD who underwent at least one month of BTKi therapy.

**Results:**

Median age at BTKi initiation was 60 years. The median duration on BTKi treatment of the whole cohort was 36.4 months. The median post-BTK survival was not reach. BTKi-based treatment was permanently discontinued in 288 (43.8%) patients during follow-up, mostly attributed to progressive disease. Within the first 6 months of BTKi treatment, 76 patients (26.3%) had early treatment discontinuation. Patients with early discontinuation had extreme worse outcome with a median post-discontinuation survival of only 6.9 months. On multivariate analysis, withdrawal BTKi by toxicity and withdrawal BTKi within 6 months retained to be independent predictors of post-BTK survival, after taking account of the response depth, lines of therapy and baseline cytogenetics including 17p deletion. The decision between BTKi monotherapy and combination therapy, along with the preference for first or second-generation BTKi, exerted no significant impact on survival.

**Discussions:**

These observations contribute valuable real-world insights into the utilization of BTKi in China. We concluded that BTKi is an effective and well-tolerated treatment for long-term use in Chinese patient population. However, it is imperative to stress that a proportion of patients discontinue BTKi early, leading to suboptimal outcomes. This study underscores the importance of adherence to BTKi therapy for improved clinical outcomes in real-world patients.

## 1 Introduction

The advent of Bruton tyrosine kinase inhibitors (BTKi) revolutionized the management of patients with B-cell lymphoproliferative diseases (BLPD), especially for chronic lymphocytic leukemia (CLL), Waldenstrom macroglobulinemia/lymphoplasmacytic lymphoma (WM/LPL) and mantle cell lymphoma (MCL). Ibrutinib was first approved by the USA Food and Drug Administration (FDA) in 2014 for the treatment of patients with previous treated CLL. The approval was expanded to the first-line CLL setting irrespective of the patient’s 17p deletion status in 2016. Ibrutinib was approved in adults with symptomatic WM/LPL by the FDA in 2013 and the European Medicine Agency (EMA) in 2015. Furthermore, ibrutinib has been widely accepted as a standard-of-care for patients with relapsed/refractory (R/R) MCL, but it remains no consensus on the ideal timing for its introduction within the treatment algorithm (1, 2).

The use of BTKi has significantly improved the prognosis for patients with BLPD, however, one of the commonalities of this disease category is incurability. Over half of the patients relapse within five years of initiating BTKi treatment (3–5). Given the indolent nature and extended survival of BLPD patients, choosing a treatment regimen must consider the delicate balance between efficacy and tolerability. The pivotal role of BTKi is undisputed, however, there are ongoing questions for its real-world usage as follows.

First, patients involved in clinical trials are under close scrutiny and are highly selected, therefore, they may not fully embody the real-world treatment dynamics. In China, concrete real-world evidence supporting BTKi usage is sparse. Second, there is a lack of real-world study that focus on the selection of different BTKi and comparing the efficiency and toxicity of BTKi among various BLPD subtypes. Additionally, it is crucial to identify the clinically relevant predictors of post-BTKi survival to guide optimal treatment decisions. Third, it is known that the incidence of BLPD is considerably lower in Asian populations compared to in Western countries, especially for CLL (6). Previous studies have suggested that Chinese CLL patients are generally younger, exhibit more mutated immunoglobulin heavy-chain variable genes (IGHV), as well as with a unique mutation landscape (7, 8). Chinese CLL patients had higher frequency mutations of *KMT2D*. *KMT2D*-mutated CLL showed impaired H3K4 methylation activity and decreased sensitivity to ibrutinib *in vitro* (7). Moreover, the ibrutinib responses in WM/LPL are affected by *MYD88* mutation status (9). There is a relatively low percentage of *MYD88* mutation in Chinese WM/LPL as reported (10, 11). These data indicate a unique biology of BLPD in Eastern populations, potentially implying a less necessity and efficacy of BTKi treatment in Chinese CLL patients. Consequently, the efficacy and clinical outcomes of BTKi treatment in Chinese patients setting needs to be further explored.

In light of these observations, real-world evidence on the efficacy and safety of BTKi on Chinese patients is important to help guide treatment planning. The principal focus of this retrospective observational study was to outline the rate of BTKi adherence, duration of BTKi exposure and reasons for discontinuation in Chinese real-world setting of BLPD. Furthermore, this study aimed to ascertain whether these factors had any impact on post-BTKi survival.

## 2 Methods

### 2.1 Patients

This is a single-center, real world, retrospective study performed at the Institute of Hematology and Blood Disease Hospital, Chinese Academy of Medical Sciences and Peking Union Medical College, diagnosed from January 2006 to October 2022. The diagnosis was established using the WHO classification criteria (12). This study included all of the patients who received at least one dose of BTKi at our hospital from January 2014 to October 2022. The specific inclusion and exclusion criteria are specified in **Supplementary Figure 1**. Discontinuation was defined as a gap of ≥90 days in treatment. Demographic data of the study cohort, lactate dehydrogenase (LDH) level, previous treatment lines, treatment regimen, adverse events (AE), and mortality data were collected. The study examined the impact of treatment duration and reasons for discontinuation on survival. Treatment response was evaluated according to standard definitions. Fluorescence *in situ* hybridization studies with specific probes for 17p13 (LSI TP53) and 11q22 (LSI ATM) and chromosome karyotype studies were performed within 6 months before starting BTKi. This study was approved by the Ethics Committee of our hospital (Institute of Hematology and Blood Disease Hospital, Tianjin, China). All patients enrolled provided written informed consent before starting treatment.

### 2.2 Outcome

Demographic and clinical data of the study cohort were evaluated with descriptive statistics. Toxicity and outcome data were collected during variable follow-up period (minimum 3 months). The response to ibrutinib therapy was assessed according to the 2014 Lugano criteria (13). Survival curves were generated by the Kaplan-Meier method and compared by using the 2-sided log-rank test. Post discontinuation survival (PDS) was defined as the period from the discontinuation of BTKi therapy until death due to any cause or until the date of the last follow-up examination. Post-BTKi overall survival (post-BTKi OS) of the patients was calculated from the first dose of BTKi to either the date of death or the date of the last follow-up examination. Post-BTKi failure-free survival (post-BTKi FFS) was defined as the interval from initial dose of BTKi to disease progression, relapse, changing treatment regimen, death, or the last follow-up evaluation.

### 2.3 Statistical analysis

Variables used for univariate analyses included: age>65, gender, line of therapy, best response, elevated LDH level, the presence of complex karyotype, the presence of 17p deletion or 11q deletion, usage of commercially available BTKi or participation in a clinical trial, the choice of first or second-generation BTKi, clinical trial participation, BTKi exposure duration and reason for discontinuation. Only those variables identified as significant at the P < 0.05 level based on univariate analysis were subsequently assessed using stepwise multivariable logistic regression. Hazard ratios (HRs) for post-BTKi survival were calculated using Cox proportional hazards models. A two-sided Fisher exact test or X-squared test were used to compare categorical parameters. Student t or Mann-Whitney U tests were used to examine differences between two continuous variables. Statistical analyses were performed using SPSS version 21.0 (IBM, Chicago, IL), Graphpad Prism 7 and R package version 3.5.1. *P*<0.05 was considered as statistically significant.

## 3 Results

### 3.1 Study population characteristics

**Figure 1:**
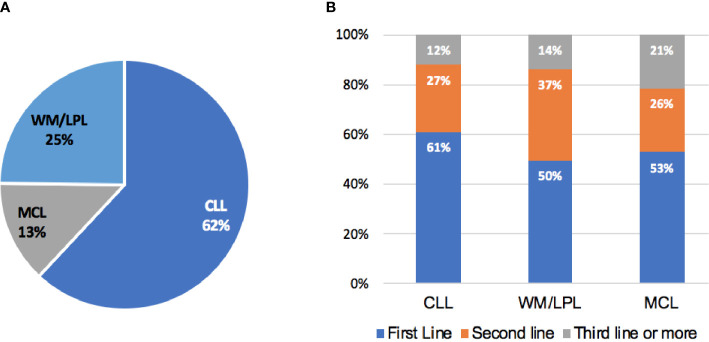
Patients distribution. **(A)** Disease distribution in patients receiving BTKi therapy. **(B)** Percentage of patients by line of therapy in which BTKi was initiated.

A total of 6177 patients with BLPD hospitalized at least once at our institute from January 2006 to October 2022. At the time of data cutoff, 673 of these patients (11.4%) were included in the study, with a median follow-up of 28.8 months from the initiation of BTKi. A flow diagram of the case selection process is presented in **Supplementary Figure 1**. The median age was 60 years, 71.0% of patients were male (**Table 1**). The most common diagnosis in BTKi treated BLPD patients was CLL (62.0%), followed by WM/LPL (28.4%), MCL (13.2%) (**Figure 1**). The cohort included 288 relapsed-refractory (R/R) and 385 treatment-naïve patients. The baseline characteristics and follow-up data for these groups were depicted in **Table 1**. The R/R group had a higher proportion of patients receiving monotherapy than previous untreated group (84% vs. 47%, *P*<0.001).

**Table 1:** Baseline demographics and clinical characteristics of patients.

Measures	Previous untreated (n=385)	Relapsed/refractory (n=288)	Total (n=673)
Median age at diagnosis, years (range)	61 (26–88)	60 (17-84)	60 (17-88)
Gender (Male: Female)	2.5/1	2.3/1	2.4/1
Disease, n (%)			
CLL	255 (65)	162 (52)	417 (62)
WM/LPL	83 (21)	84 (27)	167 (25)
MCL	47 (12)	42 (14)	89 (13)
Treatment regimen, n (%)			
Monotherapy	182 (47)	246 (84)	428 (64)
Combination therapy	203 (53)	42 (16)	245 (36)
BTKi option, n (%)			
Ibrutinib	277 (72)	174 (60)	451 (67)
Zanubrutinib	94 (24)	71 (25)	165 (25)
Orelabrutinib	9 (2)	18 (6)	27 (4)
Other	5 (1)	25 (9)	30 (4)
Elevated LDH, n (%)	105 (28)	82 (29)	187 (29)
Cytogenetics, n (%)			
17p deletion	47 (13)	36 (14)	83 (14)
11q deletion	38 (12)	30 (13)	68 (12)
Complex karyotype	76 (23)	44 (19)	120 (22)
Time, months			
Median follow-up time from BTKi initiation	26.4	34.1	28.8
Median time from diagnosis to BTKi	16.7	53.6	31.8
Median time on BTKi treatment	70.3	28.6	36.4
Median FFS post BTKi therapy	70.3	34.5	50.9
2-year survival post BTKi therapy, %	88.5	75.0	82.6
5-year survival post BTKi therapy, %	74.4	57.3	65.0
Continuation events during follow-up, n (%)	126 (33)	162 (56)	288 (43)
Death events during follow-up, n (%)	48 (12)	82 (28)	130 (19)

CLL, chronic lymphocytic leukemia; WM/LPL, Waldenstrom macroglobulinemia/lymphoplasmacytic lymphoma; MCL, mantle cell lymphoma; BTKi, Bruton tyrosine kinase inhibitors; LDH, lactate dehydrogenase; FFS, failure-free survival.

A total of 471 (70.0%) patients were treated with commercially available drug/off study. Ibrutinib was the most common choice of BTKi (451/673, 67.0%), following by zanubrutinib (24.5%) and orelabrutinib (4.0%). BTKi monotherapy was the most common regimen (Mono, 63.6%), followed by combinations of BTKi with fludarabine, cyclophosphamide and rituximab (BTKi with FCR, 13.2%), cyclophosphamide, doxorubicin, vincristine, and prednisone (CHOP)-like chemotherapy (BTKi with CHOP-like, 5.9%), rituximab only (BTKi with R, 5.6%), bendamustine rituximab (BTKi with BR, 4.9%), and other regimens (Other, 6.7%). 202 patients (30.0%) participated non-blind clinical trials, which included 13.8% investigator-initiated trials and 16.2% industry-sponsored trials. We compared the baseline characteristics of patients participating in clinical trials with those receiving commercially available BTKi. Patients in clinical trial were younger, more frequently using next generation BTKi, less likely to have elevated LDH and more likely with R/R disease compared to those using commercial BTKi (**Supplementary Table 1**).

### 3.2 BTKi discontinuation time and reason

At the last follow-up, 288 (42.8%) patients had discontinued BTKi-based treatment. The reasons for discontinuing BTKi were grouped into four categories. First, 89 of the 288 patients discontinued BTKi due to toxicity (30.9%). Among those who discontinued due to toxicity, the most common causes for discontinuation were infection (40.4%), followed by thrombocytopenia or bleeding (16.9%), skin rash (10.1%), neutropenia (9.0%), cardiac arrhythmia (9.0%), anemia (4.5%), and reactivation of hepatitis B (4.5%). Second, 140 of the 288 patients discontinued BTKi due to progression, transformation or death (48.6%). 98 of the 140 patients (70.0%) were R/R patients. Third, 11.8% patients (34/288) withdrew BTKi due to unaffordable insurance or patients’ preference. Fourth, 8.7% patients (25/288) discontinued BTKi according to the professional suggestions. Of those, 16 patients finished the treatment course of BTKi+FCR and reached minimal residual disease (MRD)-negative complete remission (CR). Following the physician’s advice, these patients discontinued BTKi and started regular post-withdrawal follow-up checks. Other patients discontinued BTKi or changed treatment regimens in preparation for surgery or allo-transplantation.

**Figure 2:**
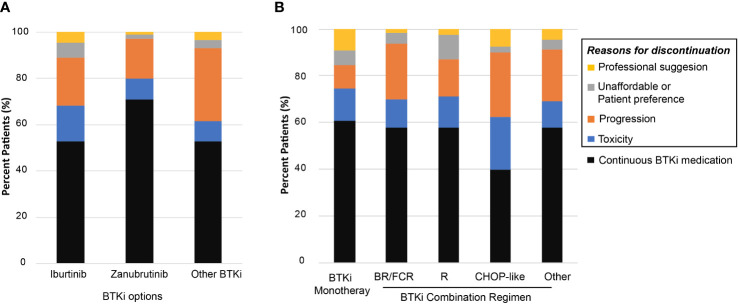
Reasons for BTKi discontinuation. **(A)** Distribution of BTKi discontinuation reasons among different BTKi option **(B)** Distribution of BTKi discontinuation reasons in patients receiving specific treatment.

**Figure 3:**
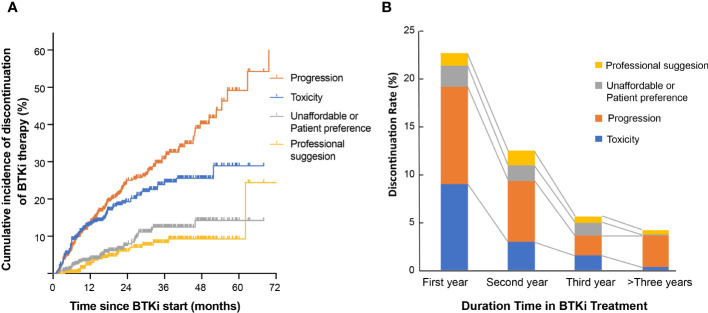
Reason for BTKi discontinuation. **(A)** Cumulative incidence of discontinuation stratified by reason for discontinuation. **(B)** The distribution of discontinuation reasons for patients who withdraw BTKi in the first year, second year, third year or who received BTKi for more than three years.

Patients treated with ibrutinib had a higher risk of discontinuing treatment during follow-up compared to those treated with zanubrutinib (47.2% vs. 29.1%, *P*<0.001, **Figure 2A**). The discontinuation difference between the two BTKi was largely due to toxicity (15.3% vs. 9.1%, *P*=0.047). The frequency of BTKi discontinuation varied by regimen, with the highest rate observed among patients treated with BTKi combined with a CHOP-like regimen (60.0% vs. 41.7% for other regimens, *P*=0.027). Patients treated with BTKi combined with BR/FCR showed a comparable rate of discontinuation than patients treated with BTKi monotherapy (**Figure 2B**). Thus, BR/FCR appeared to be relatively tolerable and effective choice as BTKi combination regimen rather than CHOP-like treatment. During the first year on BTKi, patients had a significantly higher rate of discontinuation due to toxicity compared to subsequent years (8.6% for first year, vs. 2.8% for second year, 1.5% for third year, 0.3% for fourth year or more, *P*<0.001, **Figure 3B**). Since the second year on BTKi treatment, progression had become the predominate reason for BTKi withdrawal. The cumulative incidence of discontinuation due to progression increased year by year, while the occurrence curve of other reasons-related discontinuation tended to be horizontal after the third year on BTKi treatment (**Figure 3A**).

**Figure 4:**
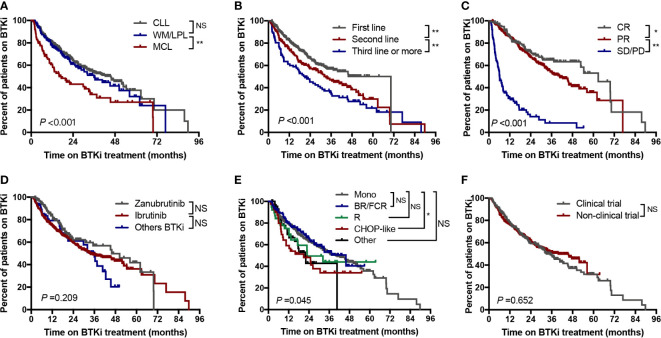
Time to BTKi discontinuation stratified by **(A)** Disease subtypes; **(B)** Line of therapy; **(C)** Depth of response; **(D)** Selectivity of BTKi; **(E)** Treatment regimen; **(F)** Clinical trial participation. CR, complete response; PR, partial response; SD, stable disease; PD, progressive disease; Mono, monotherapy; NS, no significance; *P<0.05; **P<0.01.

The median time to BTKi discontinuation of the whole cohort was 36.4 months. At 1 year, 77.2% of patients in our cohort remained on BTKi treatment, and at 2 years 60.7% remained on treatment. We also evaluated the influencing factors of duration time on BTKi in different subgroups. Among the three disease categories, patients with CLL or WM/LPL had significantly longer periods of BTKi use (45.7 and 36.0 months, respectively) compared to the patients with MCL (16.0 months) (*P*<0.001, **Figure 4A**).

Statistically, significant longer time of BTKi adherence were observed in patients with first-line therapy, those who responsed to BTKi, and those receiving monotherapy or combined R/BR/FCR treatment (**Figure 4**). Notably, there was no significant difference in BTKi adherence time by monotherapy group *versus* combination therapy group (**Supplementary Figure 2**). The duration of BTKi treatment remained similar when comparing the choice of different generations of BTKi (**Figure 4D**) and and between clinical trial participation versus commercial use (**Figure 4F**).

### 3.3 Response to treatment and outcome

The best overall response rate (ORR) of the entire cohort was 82.8%, with a CR rate of 20.2%. The combination therapy group showed significantly higher CR rates than the monotherapy group (37.1% vs. 11.0%, *P*<0.001), however the ORR rate was similar between the two groups (85.3% vs. 83.9%, *P*=0.620). The CR rate was higher in untreated subgroup than relapse/refractory group (27.8% vs. 11.1%, P<0.001). The median time from diagnosis to BTKi starting was 31.8 months.

**Figure 5:**
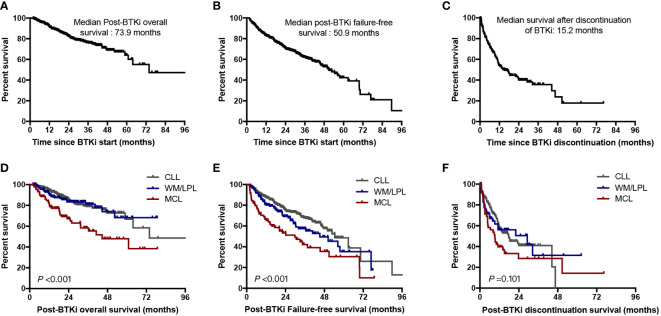
Outcomes for the entire cohort. **(A)** Overall survival for the whole cohort of patients since BTKi treatment start; **(B)** Failure-free survival since BTKi treatment start; **(C)** Overall survival from date of BTKi discontinuation; **(D)** Post-BTKi overall survival by disease subtypes; **(E)** Post-BTKi failure-free survival by disease subtypes; **(F)** Post-BTKi discontinuation survival by disease subtypes.

With a median follow-up time of 28.8 months, 221 patients (77 in first-line group, and 144 in previously treated group) had experienced BTKi treatment failure. The estimated median post-BTKi FFS of the entire cohort was 50.9 months, with 2-year and 5-year FFS of 70.8% and 42.3%, respectively (**Figure 5A**). The median post-BTKi OS was not reached, with 2-year and 5-year OS of 82.6% and 65.0%, respectively (**Figure 5B**). Inferior outcome was observed in patients who discontinued BTKi by any cause. The median PDS was 15.2 months, which means that more than half of the patients died within 2 years after discontinuation of BTKi (**Figure 5C**). According to the variety of disease in patients on BTKi treatment, we found patients with CLL and WM/LPL had superior post-BTKi survival than patients with MCL (*P*<0.001, **Figure 5D**). Different diseases had distinct timeframes to BTKi failure, with median post-BTKi FFS of 54.5 months in CLL, 45.2 months in WM/LPL, 29.8 months in MCL (*P*<0.001, **Figure 5E**). This variation in the efficacy of BTKi across different disease subtypes aligns with previous clinical trial findings (**Figures 5D–F**).

**Figure 6:**
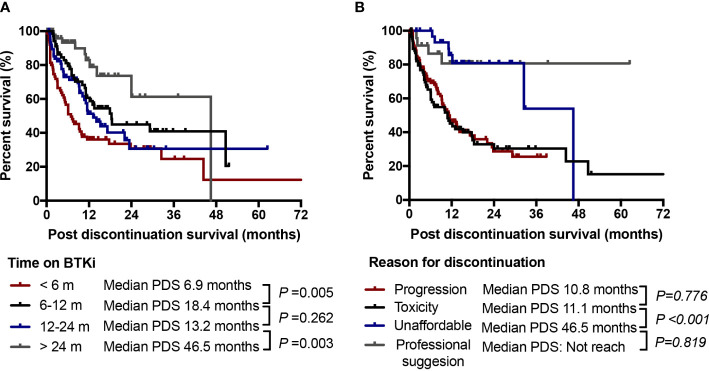
Discontinuation time and reason. **(A)** Post discontinuation survival (PDS) of patients according to time of duration on BTKi treatment; **(B)** PDS of patients according to the cause of discontinuation. NS, no significance.

Among the 288 patients who had discontinued BTKi during follow-up, 26.4% of those discontinued within 6 months on BTKi identified as early discontinuation. The major reason for early discontinuation was toxicity (48.7%), followed by disease progression (42.1%). Patients with early discontinuation had extreme worse outcome with a median PDS of only 6.9 months. While patients who discontinued after 24 months on BTKi had a significantly longer survival whatever the withdrawal cause (median PDS: 46.5 months vs. 13.2 months for those discontinued in 12-24 months, P=0.003, **Figure 6A**). When we looked into the effect of discontinuation reason on survival, patients who discontinued due to toxicity had similar post discontinuation survival to those who discontinued due to disease progression (median PDS 10.8 and 11.1 months, *P*=0.776). Patients who withdrew BTKi due to economic reasons or professional suggestion had relatively longer survival after BTKi discontinuation compared to other reasons (median PDS 46.5 and not reach, respectively, **Figure 6B**). In our subgroup analysis of specific disease subtypes and treatment statuses, we observed that patients with CLL and WM/LPL, as well as both treatment-naïve and relapsed/refractory patients, who discontinued BTKi due to toxicity or within 6 months had significantly shorter post-BTKi survival (**Supplementary Figure 3**). However, the reason for discontinuation held no prognostic significance in patients with MCL (**Supplementary Figure 3E**).

### 3.4 Prognostic factors of post-BTKi survival

Patients were stratified by BTKi regimen (monotherapy versus combination therapy), choice of BTKi (Ibrutinib versus Zanubrutinib versus others), line of therapy (front-line versus R/R), depth of response (CR versus PR versus less than PR), clinical trial participation versus commercial use, reasons for discontinuation (intolerance versus progressive disease versus other reasons), and timing of discontinuation events (within 6 months versus more than 6 months). Besides, we also analyzed the impact of clinical characteristics on post-BTKi survival, such as age, LDH level and cytogenetic abnormalities.

**Figure 7:**
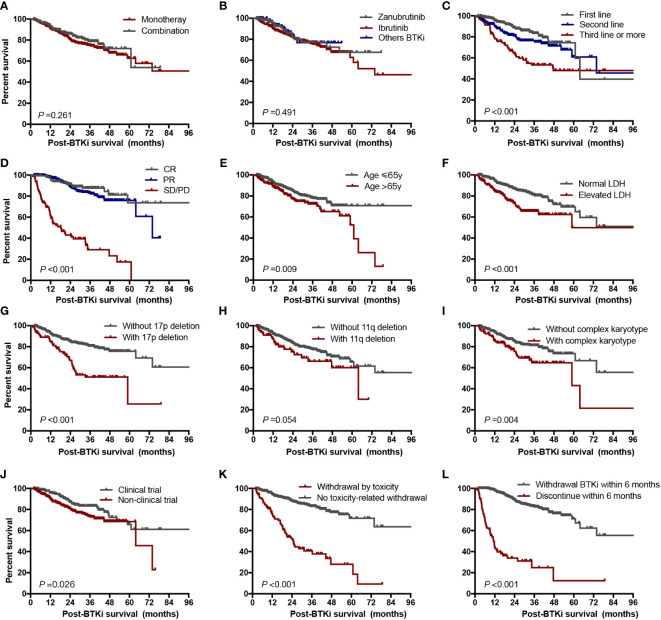
Post-BTKi survival according to prognostic factors. Overall survival after start of BTKi treatment stratified by treatment regimen **(A)**, selectivity of BTKi **(B)**, line of therapy **(C)**, depth of response **(D)**, age **(E)**, LDH level **(F)**, 17p deletion status **(G)**, 11q deletion status **(H)**, karyotype status **(I)**, clinical trial participation **(J)**, reason of discontinuation **(K)**, and duration time on BTKi **(L)**. LDH, lactate dehydrogenase.

We found line of therapy, depth of response less than PR, elevated LDH level with complex karyotype, with 17p deletion, withdrawal BTKi by toxicity and withdrawal BTKi within 6 months were associated with inferior post BTKi FFS and OS (**Supplementary Figure 4**; **Figure 7**). Besides, the following variables were also associated with inferior post BTKi OS: age>65 and commercial BTKi use other than participating a clinical trial (**Figure 7**). However, no significant impact on outcome was identified among patients receiving different generations of BTKi (*P*=0.491, **Figure 7B**). Similar conclusions were reached when we considered only patients with CLL (**Supplementary Figure 5**). Besides, patients with combination had a trend of better post-BTKi FFS than those with monotherapy therapy but with no statistical significance (*P*=0.058).

To determine the independent factors associated with post BTKi survival, we included all the factors significant in univariate analyses in a multivariate Cox model. Finally, category of disease (hazard ratio [HR] = 1.8, *P*=0.021), age>65 (HR = 1.6, *P*=0.045), with 17p deletion (HR = 2.7, *P*<0.001), not first-line of therapy (HR = 2.3, *P*<0.001), withdrawal BTKi by toxicity (HR = 2.4, *P*<0.001) and withdrawal BTKi within 6 months (HR = 6.4, *P*<0.001) were independent predictors of inferior post-BTKi survival (**Table 2**).

**Table 2:** Multivariate regression models evaluating risk factors for overall survival after start of BTKi treatment.

Variable	HR (95% CI)	*P* value
Category of disease (MCL vs. CLL/WMLPL)	1.8 (1.1-3.1)	**0.021**
Age (>65 vs. ≤65)	1.6 (1.0-2.4)	**0.045**
LDH (Elevated vs. Normal)	1.4 (0.9-2.2)	0.145
Complex karyotype (Yes vs. No)	1.2 (0.8-1.9)	0.369
17p deletion (Yes vs. No)	2.7 (1.7-4.3)	**0.001**
Line of therapy (Non-first line vs. First line)	2.3 (1.5-3.6)	**0.001**
Best response of BTKi (CR vs. PR/SD/PD)	0.6 (0.3-1.2)	0.167
Clinical trial participation (Yes vs. No)	0.7 (0.4-1.2)	0.177
Withdrawal BTKi by toxicity (Yes vs. No)	2.4 (1.4-3.9)	**0.001**
Withdrawal BTKi within 6 months (Yes vs. No)	6.4 (4.0-10.3)	**0.001**

Significant P values were marked in bold. BTKi, Bruton tyrosine kinase inhibitors; CLL, chronic lymphocytic leukemia; WM/LPL, Waldenstrom macroglobulinemia/lymphoplasmacytic lymphoma; MCL, Mantle cell lymphoma HR, Hazard ratio. LDH, Lactate dehydrogenase; CR, Complete response; PR, Partial response; SD, Stable disease; PD, progressive disease.

## 4 Discussion

This study outlines the impact of BTKi exposure duration and reasons for discontinuation on survival in patients with BLPD. To the best of our knowledge, this series is the most comprehensive report so far on BTKi-treated patients in a clinical setting in China. Our observations showed a post-BTKi FFS of 70.3 months for untreated CLL and 40.6 months for R/R CLL. This is slightly shorter than that previously reported in clinical trials where the median progression-free survival (PFS) was not reach in untreated CLL [RESONATE-2 cohort (14)] and was 44.5 months in R/R CLL [RESONATE cohort (4)]. Nevertheless, the outcome in our cohort align with the results of the real-world analysis from other countries, such as US, UK, Denmark (15–17) (**Supplementary Table 2**). These findings suggest that outcomes in real-world clinical practice may be less favorable when compared to those from clinical trial patients. In our cohort, 30 percent of patients participated the BTKi-related non-blind clinical trials. The preference of first or second-generation BTKi was different, while more patients took ibrutinib in commercial use, and more patients took zanubrutinib and other BTKi in clinical trial (**Supplementary Table 1**). It is interesting to find that adherence time on BTKi treatment and post-BTKi FFS was similar in the two groups. But patients in clinical trial showed superior post-BTKi survival than those in commercial usage (**Figure 7J**, median survival 63.8 months vs. not reach, *P*=0.026). However, this outcome difference no longer existed in the following multivariate analysis. This superior survival in clinical trials could, in part, be attributed to a more specialized supervision and a lower incidence of comorbid patients. Although random clinical trials remain to be gold standard for evidence-based medicine, real-world evidence is crucial to bridging knowledge gaps and guide decision-making in regular clinical practice.

In spite of the outcome in our study were comparable with other real-world studies, the reasons for discontinuation were quite different in our setting. With a median follow-up of 28.8 months, the overall discontinuation of BTKi was 42.8%, which is consistent with both real-world and clinical trial studies. However, the most common reason for discontinuation in our cohort was disease progression in both first-line group and R/R group. This contrasts with clinical trial and other real-world studies where the majority of patients discontinue were due to toxic toxicity (18–20). In the long-term follow-up data of RESONATE-2 study, 41% discontinued ibrutinib treatment; of these, 21% discontinued by AE, and only 6% discontinued by progression (14). Similarly, in a real-world US setting, 41% of CLL patients (n=616) discontinued ibrutinib, and toxicity was the most common reason for discontinuation in all settings, accounting for 63.1% of discontinuations in front-line use and 50.2% in R/R use (15). This disparity could be attributed to the relatively poorer compliance of Chinese patients, resulting in dose reductions and temporary discontinuation more commonly occurred. Therefore, fewer intolerance-related discontinuations and more progression were observed in our cohort. On the other hand, most discontinuation events due to toxicity occurred within the first year on BTKi. But the cumulative incidence of progression went up year by year. Thus, the discrepancy could also be explained by a longer follow-up time in our cohort. In addition, this inter-study difference may in part be due to the biological and genotype variations between Chinese and Western BLPD patients.

Patients exhibited rapid disease progression following the discontinuation of BTKi treatment. We found patients who discontinued BTKi due to toxicity had comparably dismal outcome to those discontinued due to progression, in line with a prior real-world study (16). However, this was in contrast with prior studies reporting inferior OS for patients with progressing on ibrutinib compared with patients discontinuing due to AEs (15, 21). This inter-study discrepancy may partially be explained by improved later-line treatment options with other targeted agents such as venetoclax upon progression on BTKi. In addition, we identified the initial 6 months of BTKi treatment as critical, but the majority of discontinuations due to AEs appear in this time period. This could partially elucidate the exceedingly poor outcome in patients who discontinued due to toxicity. As a consequence, we need to precisely select patients who can tolerate and derive the most benefit from BTKi treatment. To this end, we conducted one of the most comprehensive studies to identify the independent factors in predict of post-BTKi survival. A multivariate cox model was developed taking account of demographic data, clinical characteristics, cytogenetic abnormalities, treatment patterns, treatment response, as well as reasons and timing for discontinuation. As expected, age>65 and 17p deletion were independent predictors of inferior survival following BTKi treatment. Similar findings regarding del(17p) patients were reported in a 3-year follow-up multicenter study (22), as well in the RESONATE-17 study (23). Interestingly, discontinuation due to toxicity and discontinuation within 6 months on BTKi remained predictive markers for survival when other prognostic markers were considered. Regardless of the disease subtype, number of therapy lines or presence of 17p deletion, patients with non-relapse discontinuation or early discontinuation of BTKi experienced significantly dismal outcome. These findings emphasized the importance of maintaining high adherence to BTKi. Complications, physical fitness status as well as financial barriers needs to be fully considered before initiation of BTKi treatment.

It is noteworthy that this study explored the effects of BTKi exposure duration and discontinuation reasons on survival in patients with BLPD. BTKi has become the routine clinical practice for untreated and R/R CLL and WM/LPL. While BTKi monotherapy was not one of the front-line treatments recommended by the NCCN guidelines for untreated MCL (24), it is important to note that frequent use is observed in studies leveraging real-world data. Even though the relatively high proportion of front-line BTKi usage in our MCL cohort, patients with MCL still showed markedly dismal outcome than those with CLL or WM/LPL. Therefore, in order to reduce the influence of disease variation on our conclusion, we included the category of disease into the multivariate analysis. We found the impact of the timing and reason of BTKi discontinuation on survival remained significant after adjusting for disease category.

In addition to evaluating real-world adherence to BTKi, we also discussed the impact of the selection of different BTKi. Currently, ibrutinib is most commonly used BTKi. However, ibrutinib is associated with AE attributed to off-target effects in at least 20% of the patients, such as hemorrhage, atrial fibrillation, ventricular arrhythmias, and hypertension (25, 26). More recently, the next generation of BTKi such as Zanubrutinib and orelabrutinib have become available in clinical practice in China. The phase 3 ALPINE study showed a superior ORR and improved PFS in patients with R/R CLL treated with zanubrutinib compared with ibrutinib (27). In real-world investigation, we found patients with zanubrutinib had noticeably fewer toxicities and a lower risk of drug withdrawal than those on ibrutinib. However, the median adherence time on BTKi treatment was similar, and we did not obverse a significant difference in post-BTKi FFS or OS between the two groups. We arrived at the same conclusion when we only took account of patients with CLL. This might partially be explained by the higher proportion of R/R disease in those who took zanubrutinib than those with ibrutinib (43.0% vs. 38.6%), but the difference is not significant (*P*=0.318). Nevertheless, it’s important to note that the retrospective nature of the study might introduce selection bias. In addition, it is difficult to collect data on dose adherence data in real-world practice. We only focused on the impact of discontinuation, while the influence of dose reduction was not addressed. Due to these limitations, the conclusion should be interpreted cautiously.

We have demonstrated the real-world treatment patterns of BTKi. Prior clinical trials have reported promising efficacy with BTKi combination therapy (28–30). However, in this study, the combination therapy group demonstrated better CR rates but similar ORR rate and relatively higher rate of toxicity. As a consequence, we observed no difference of survival outcome between the combination and monotherapy groups. It is important to carefully consider the patient tolerance and drug discontinuation before making decision of BTKi combination regimen.

## 5 Conclusions

In conclusion, despite higher rates of discontinuation than anticipated, our study demonstrated that BTKi is an effective and well-tolerated treatment for long-term use in Chinese patient population. Early discontinuation and withdrawal BTKi by toxicity were confirmed to be predictors of post-BTK survival, independent of response depth, lines of therapy and baseline cytogenetics including 17p deletion. Further investigation is necessary to identify patients at high risk of poor adherence, and better guide optimal individualized treatment decisions.

## Data availability statement

The original contributions presented in the study are included in the article/**Supplementary Material**. Further inquiries can be directed to the corresponding authors.

## Ethics statement

The studies involving human participants were reviewed and approved by Institute of Hematology & Blood Diseases Hospital, Chinese Academy of Medical Sciences & Peking Union Medical College. The patients/participants provided their written informed consent to participate in this study.

## Author contributions

SY and LQ designed the study and provided leadership. YYa analyzed the data and wrote the manuscript. RL, SY, TW, YYu, QW, YYa, WS, WH, DZ, and GA managed patients and collected samples. YH, YL, and WX performed clinical data annotation. LQ, SY, and JW were responsible for checking diagnosis. All authors reviewed the manuscript and provided final approval for submission.

## Supplementary material

Supplementary Figure 1 Patient attrition flowchart. BLPD, B-cell lymphoproliferative diseases; CLL, chronic lymphocytic leukemia; WM/LPL, Waldenstrom macroglobulinemia/lymphoplasmacytic lymphoma; MCL, Mantle cell lymphoma.

Supplementary Figure 2 Time on BTKi treatment stratified by treatment regimen: BTKi monotherapy or BTKi combined with other regimens.

Supplementary Figure 3 Post-BTKi survival according to disease subtypes and treatment statuses. Post-BTKi survival stratified by reason of discontinuation in CLL patients **(A)**, MCL patients **(B)**, WM/LPL patients **(C)**, treatment-naïve patients **(G)** and relapsed/refractory patients (I); Post-BTKi survival stratified by duration time on BTKi in CLL patients **(D)**, MCL patients **(E)**, WM/LPL patients **(F)**, treatment-naïve patients **(H)** and relapsed/refractory patients **(J)**.

Supplementary Figure 4 Post-BTKi failure-free survival (FFS) according to prognostic factors. FFS after start of BTKi treatment stratified by age **(A)**, clinical trial participation **(B)**, LDH level **(C)**, karyotype status **(D)**, 17p deletion status **(E)**, 11q deletion status **(F)**, treatment regimen **(G)**, depth of response **(H)**, line of therapy **(I)**. LDH, lactate dehydrogenase.

Supplementary Figure 5 Time on BTKi treatment **(A)**, post-BTKi failure-free survival **(B)** and post-BTKi overall survival **(C)** stratified by the selectivity of BTKi in patients with CLL.
