# Supplementary material for: Real-world treatment patterns, discontinuation and clinical outcomes in patients with B-cell lymphoproliferative diseases treated with BTK inhibitors in China
Source: Front Immunol. 2023 Jul 7;14:1184395. doi: 10.3389/fimmu.2023.1184395 (PMC10360166; doi:10.3389/fimmu.2023.1184395)
Supplement: Supplementary Figure 1 — Patient attrition flowchart. BLPD, B-cell lymphoproliferative diseases; CLL, chronic lymphocytic leukemia; WM/LPL, Waldenstrom macroglobulinemia/lymphoplasmacytic lymphoma; MCL, Mantle cell lymphoma. [file Image_1.pdf]

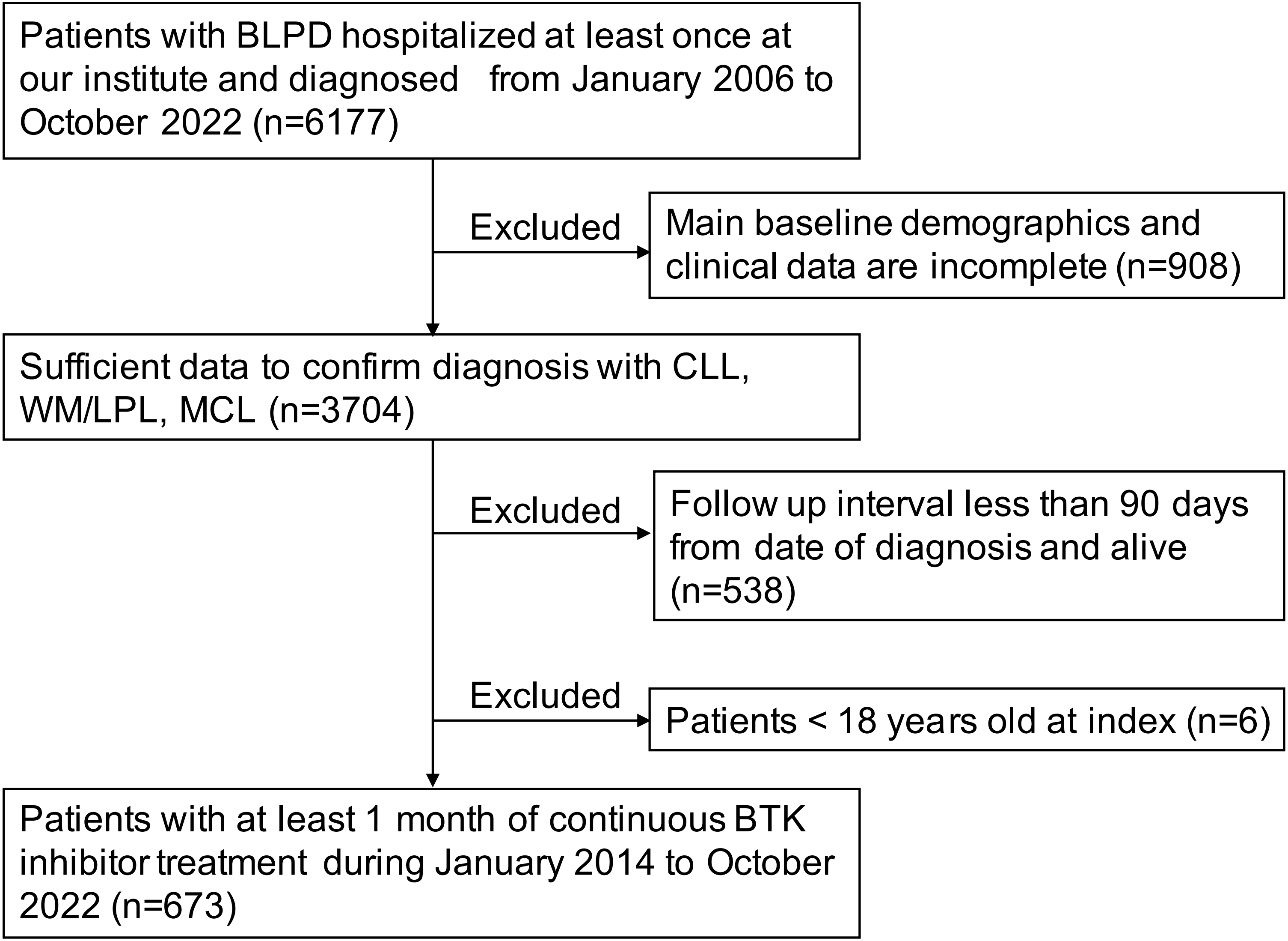
Patients with BLPD hospitalized at least once at our institute and diagnosed from January 2006 to October 2022 (n=6177)
Excluded
Main baseline demographics and clinical data are incomplete (n=908)
Sufficient data to confirm diagnosis with CLL, WM/LPL, MCL (n=3704)
Excluded
Follow up interval less than 90 days from date of diagnosis and alive (n=538)
Excluded
Patients < 18 years old at index (n=6)
Patients with at least 1 month of continuous BTK inhibitor treatment during January 2014 to October 2022 (n=673)
