# Supplementary figures and images for: Real-world treatment patterns, discontinuation and clinical outcomes in patients with B-cell lymphoproliferative diseases treated with BTK inhibitors in China
Source: Front Immunol. 2023 Jul 7;14:1184395. doi: 10.3389/fimmu.2023.1184395 (PMC10360166; doi:10.3389/fimmu.2023.1184395)

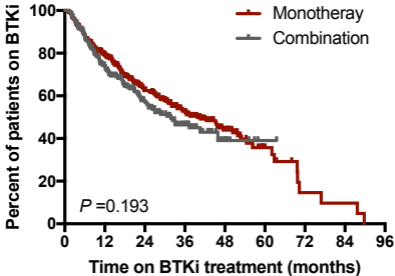

Supplement: Supplementary Figure 2 — Time on BTKi treatment stratified by treatment regimen: BTKi monotherapy or BTKi combined with other regimens. [file Image_2.pdf]

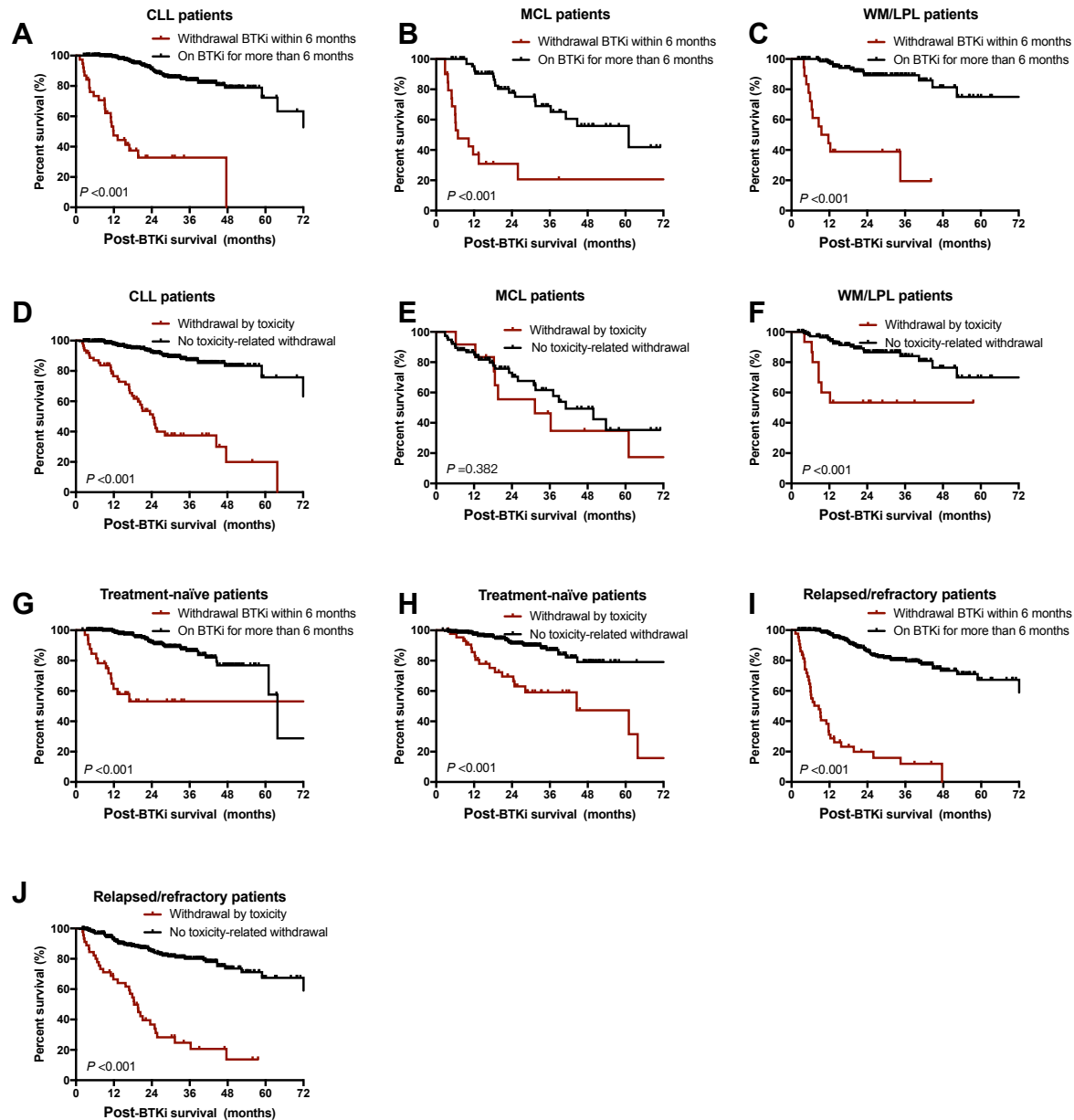

Supplement: Supplementary Figure 3 — Post-BTKi survival according to disease subtypes and treatment statuses. Post-BTKi survival stratified by reason of discontinuation in CLL patients (A), MCL patients (B), WM/LPL patients (C), treatment-naïve patients (G) and relapsed/refractory patients (I); Post-BTKi survival stratified by duration time on BTKi in CLL patients (D), MCL patients (E), WM/LPL patients (F), treatment-naïve patients (H) and relapsed/refractory patients (J). [file Image_3.pdf]

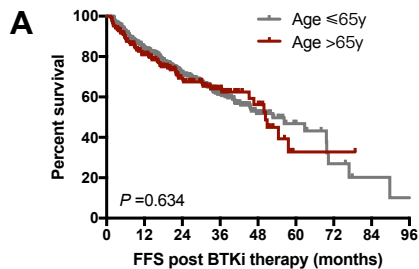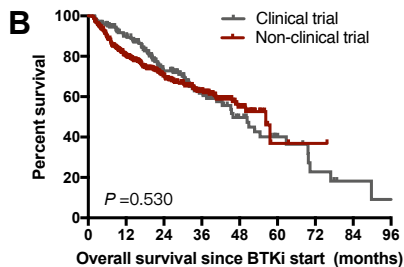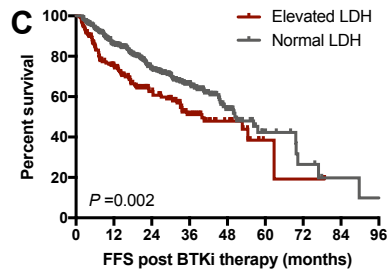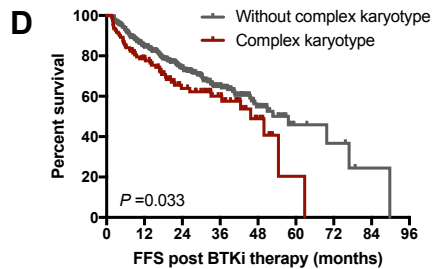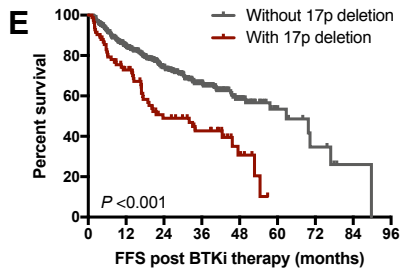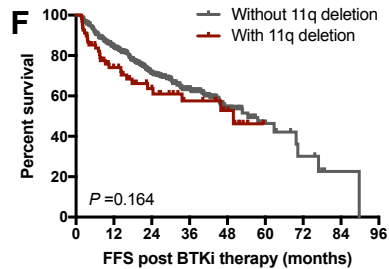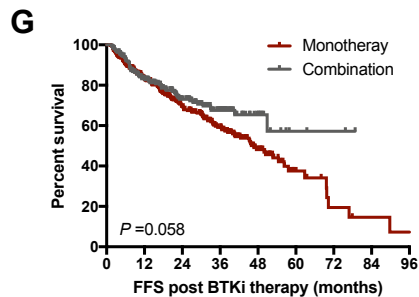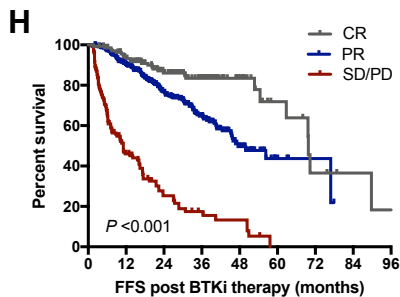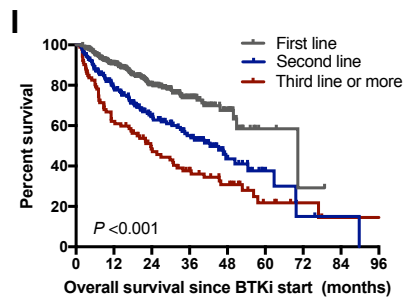

Supplement: Supplementary Figure 4 — Post-BTKi failure-free survival (FFS) according to prognostic factors. FFS after start of BTKi treatment stratified by age (A), clinical trial participation (B), LDH level (C), karyotype status (D), 17p deletion status (E), 11q deletion status (F), treatment regimen (G), depth of response (H), line of therapy (I). LDH, lactate dehydrogenase. [file Image_4.pdf]

**A**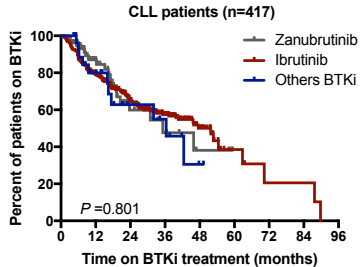**B**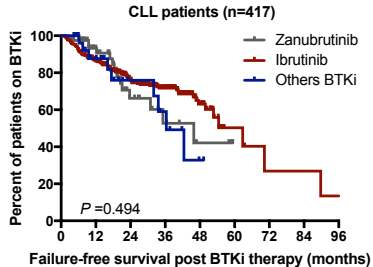**C**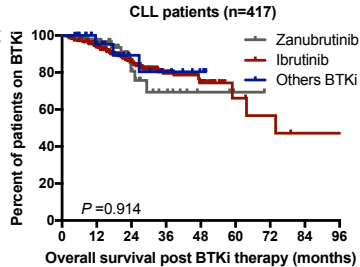

Supplement: Supplementary Figure 5 — Time on BTKi treatment (A), post-BTKi failure-free survival (B) and post-BTKi overall survival (C) stratified by the selectivity of BTKi in patients with CLL. [file Image_5.pdf]
