# Supplementary material for: Real-world treatment patterns, discontinuation and clinical outcomes in patients with B-cell lymphoproliferative diseases treated with BTK inhibitors in China
Source: Front Immunol. 2023 Jul 7;14:1184395. doi: 10.3389/fimmu.2023.1184395 (PMC10360166; doi:10.3389/fimmu.2023.1184395)
Supplement: Supplementary file 6 [file Table_1.pdf]

Supplementary table 1. The difference of baseline characteristics between patients who participating clinical trial and patients with commercial BTKi use. NS. No significance

Measures	Commercial use (n=471)	Clinical trial (n=202)	*P* value
Median age at diagnosis, years (range)	61 (17-88)	58 (26-84)	0.004
Gender (M:F)	2.4/1	2.5/1	NS.
Disease, n (%)			NS.
CLL	297 (63)	120 (59)	
WM/LPL	110 (23)	57 (28)	
MCL	64 (14)	25 (12)	
Treatment regimen, n (%)			NS.
Monotherapy	306 (65)	122 (60)	
Combination therapy	165 (35)	60 (40)	
BTKi option, n (%)			<0.001
Ibrutinib	369 (78)	82 (41)	
Zanubrutinib	88 (19)	77 (38)	
Orelabrutinib	14 (3)	13 (6)	
Other	0 (0)	30 (15)	
Elevated LDH, n (%)	148 (33)	39 (20)	0.004
Cytogenetics, n (%)			
17p deletion	63 (14)	20 (12)	NS.
11q deletion	51 (13)	17 (11)	NS.
Complex karyotype	87 (22)	33 (20)	NS.
Line of therapy, n (%)			0.026
First line	281 (60)	104 (51)	
Second line	136 (29)	60 (30)	
Third line or more	54 (11)	38 (19)	
