# Supplementary material for: Real-world treatment patterns, discontinuation and clinical outcomes in patients with B-cell lymphoproliferative diseases treated with BTK inhibitors in China
Source: Front Immunol. 2023 Jul 7;14:1184395. doi: 10.3389/fimmu.2023.1184395 (PMC10360166; doi:10.3389/fimmu.2023.1184395)
Supplement: Supplementary file 7 [file Table_2.pdf]

Supplementary table 2. The timing of BTKi discontinuation and median progression-free survival in different CLL cohorts.

Cohort		Patients	Percent of patients on BTKi			Median PFS, months
			At 1 year	At 2 years	At 3 years	
Clinical trails	RESONATE cohort [1]	R/R CLL n = 195	81%	69%	53%	44.5
	RESONATE-2 cohort [2]	Untreated CLL n = 269	93%	87%	73%	NR, 70% at 5 years
Real-world studies	Australian real-world cohort (PBS) [3]	R/R CLL	85%	74%	64%	-
	US real-world cohort [4]	Mainly R/R CLL (87%) n=616	-	59% at 17 months	-	35.0
	Denmark real-world cohort [5]	Mainly R/R CLL (81%) n= 205	75.2%	63.8%	-	41.2
	Chinese real-world cohort (our cohort)	R/R CLL n = 162	74%	54%	43%	40.6
	Chinese real-world cohort (our cohort)	Untreated CLL n = 255	85%	71%	62%	70.3

Reference

[1] Munir T, Brown JR, O'brien S, et al. Final analysis from RESONATE: Up to six years of follow-up on ibrutinib in patients with previously treated chronic lymphocytic leukemia or small lymphocytic lymphoma[J]. American Journal of Hematology, 2019, 94(12).

[2] Burger JA, Barr PM, Robak T, et al. Long-term efficacy and safety of first-line ibrutinib treatment for patients with CLL/SLL: 5 years of follow-up from the phase 3 RESONATE-2 study[J]. Leukemia, 2020, 34(3): 787-798. doi:10.1038/s41375-019-0602-x.

[3] Mulligan SP, Opat S, Marlton P, et al. Ibrutinib use, treatment duration, and concomitant medications in Australian patients with relapsed or refractory chronic lymphocytic leukaemia[J]. Br J Haematol, 2022, 198(4): 790-793. doi:10.1111/bjh.18306.

[4] Mato AR, Nabhan C, Thompson MC, et al. Toxicities and outcomes of 616 ibrutinib-

treated patients in the United States: a real-world analysis[J]. Haematologica, 2018, 103(5): 874-879. doi:10.3324/haematol.2017.182907.

[5] Aarup K, Rotbain EC, Enggaard L, et al. Real-world outcomes for 205 patients with chronic lymphocytic leukemia treated with ibrutinib[J]. Eur J Haematol, 2020, 105(5): 646-654. doi:10.1111/ejh.13499.
